# Preconceptions influence women’s perceptions of information on breast cancer screening: a qualitative study

**DOI:** 10.1186/s13104-015-1327-1

**Published:** 2015-09-03

**Authors:** Mikael Johannes Vuokko Henriksen, Ann Dorrit Guassora, John Brodersen

**Affiliations:** Research Unit and Section for General Practice, Department of Public Health, University of Copenhagen, Oster Farimagsgade 5, 1014 Copenhagen K, Denmark; Centre for Clinical Education 5404, University of Copenhagen and the Capital Region of Denmark, Copenhagen, Denmark; Research Unit for General Practice, Region Zealand, Denmark

**Keywords:** Informed consent (MeSH), Cognitive dissonance (MeSH), Mass screening (MeSH), Mammography (MeSH), Health communication (MESH)

## Abstract

**Background:**

Screening for breast cancer has been subject to intense debate in recent decades regarding benefits and risks. Participation in breast cancer screening should be based on informed choice, and most countries approach this by sending information leaflets with invitations to attend screening. However, very little attention has been paid to the decision-making process and how the information leaflets are used and understood by women. The aim of this study is twofold. First, we use a theoretical framework to explore how the framing of information influences the intention to participate in breast cancer screening. Second, we discuss how information and attitudes held prior to receiving the invitation influence the perception of the balance between the benefits and risks harms of screening.

**Methods:**

We used a qualitative design and interviewed six women who were soon to receive their first invitation to participate in the breast screening programme in Denmark. The selected women received a copy of the official information leaflet 1 week before we interviewed them. The six women were interviewed individually using an interview guide based on the theory of planned behaviour. We used meaning condensation for our initial analysis, and further analysis was guided by the theory of cognitive dissonance.

**Results:**

For our participants, the decision-making process was dominated by the attitudes of the women’s circle of acquaintances and, to a lesser extent, by the information that accompanied the screening invitation. Information that conflicted with attitudes the women already held was actively disregarded. The risk of overdiagnosis as a potentially harmful effect of participation in mammography screening was unknown to the women in our study. An isolated framing effect was not found.

**Conclusion:**

Women have expectations about breast cancer screening that are formed before they receive information from the screening programme. These expectations compromise the perception of balance between screening benefits and potential harmful effects. They also influence the perception of the information in the breast screening leaflet. The phenomenon of overdiagnosis is unknown to the women.

## Background

Both ethics and the law require that all health interventions are preceded by informed consent, screening for cancer being no exception. The aim of informed consent is to respect autonomy and to ensure that no person is deceived about or coerced into medical interventions. Accordingly, individuals should have access to whatever information they need in order to make an informed decision [1]. As screening programmes generally invite healthy individuals to participate, the need to inform participants about uncertainties regarding benefits and potential harmful effects is even greater [2, 3].

There has been vigorous debate in recent decades about evidence from breast cancer screening and the balance between the intended benefits [4] in terms of mortality reduction, and unintended harmful effects in terms of false-positive findings [5, 6], overdiagnosis, and overtreatment [7]. The Independent UK Panel on Breast Cancer Screening concluded: “*screening for breast cancer reduces breast cancer mortality but that some overdiagnosis occurs*.” The Independent UK Panel recommends that information about the effectiveness of the screening programme and the risk of overdiagnosis should be more clearly communicated to the women invited to participate [8].

The general public’s knowledge on the effect of screening programmes is scanty, and research suggests that only 1.5 % of Europeans know the actual benefits of participating in breast cancer screening [9]. Further, the risk of overdiagnosis is unknown to the women invited for screening [10].

Most countries present the facts about potential benefits and harmful effects in information leaflets accompanying the official invitation to attend a screening programme. However, studies have identified a lack of information, particularly on the potential risks [11, 12]. In addition, the information leaflets have been criticised for being non-neutral in favour of participation, nudging women to feel confident about taking part at the expense of an objective balance [13]. These studies have focused on the information provided by the screening programmes, but according to the concept of informed consent, the focus should be on the women’s understanding of the information and the role it plays in building an understanding of the balance between benefits and harms.

Decision making is not only dependent on the content of the information, it also relies on the way the information is presented, and both aspects will influence the individual’s decisions [14] and choices about whether or not to attend cancer screening programmes [15]. When information about an intervention for a potentially deadly disease such as breast cancer is presented to women, the use of framing to describe the outcome is of specific interest. For example, the same information can be framed in terms of mortality or in terms of survival. Furthermore, information leaflets are one among several sources available to women that assist them in decision making. Many women may hold individual perceptions about the intervention that is presented to them. Women’s decision making might be explained by the theory of planned behaviour (TPB) [16], which describes aspects of attitude, subjective norms, and self-control in the formation of health intentions (see “Theory” section).

The challenge of minimizing the gap between the public’s perception and the available evidence about benefits and harmful effects of screening seems to be not just a matter of consensus on what information to include. To secure informed consent, the perception of the information presented to women invited to participate in breast cancer screening should be further investigated. This investigation should include the effect of how facts are framed and illuminate the context in which the women base their interpretations of the information.

The aim of this study, therefore, is to use a theoretical framework to explore how the framing of information influences the intention to participate in breast cancer screening. We look at how information received or attitudes held prior to the invitation to attend screening influence the perception of information about the balance between benefits and harmful effects.

## Theory

**Fig. 1:**
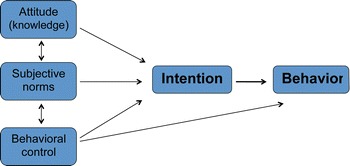
The theory of planned behaviour [16]. *Legend* a conceptual representation of the elements of the Theory of Planned Behaviour described by Ajzen

To identify and characterise the context of informed consent regarding screening mammography, we chose Ajzen’s well-established TPB [16]. TPB explains factors that contribute to a person’s intentions regarding their behaviour, and is readily applicable to health behaviour. The model describes intention based on ‘attitude’, ‘subjective norms’ and ‘perceived behavioural control’ (Fig. 1). ‘Attitude’ covers the individual’s perception of the effect of the health behaviour, in this case having a screening mammogram. This perception is combined with a personal evaluation of the outcome. The ‘subjective norms’ encompass a combination of the expectation of other people’s reaction towards the action, weighted against the motivation to comply with these people’s opinions. ‘Perceived behavioural control’ describes the weighting between the presence or absence of barriers, and the influence the individual believes a barrier will have on the ability to implement the behaviour. For example, the distance to the mammography clinic may be perceived as a barrier and this will affect the individual’s belief as to whether getting to the clinic is feasible. The interaction between these factors varies between populations and health behaviours.

We used TPB as a framework for the interview guide and we asked about and recorded sources of information about mammography screening and the women’s attitudes towards these sources.

The theory of cognitive dissonance by Festinger describes how people strive towards internal consistency and how feelings of tension and uncertainty develop when individuals are confronted with information that contradicts their personal beliefs and attitudes [17]. People will usually strike towards consistency in the effort to avoid a psychological inconsistency, which Festinger named dissonance. A heavy smoker, for example, would be expected to feel dissonance if s/he encounters information stating that smoking is unhealthy. The individual has different strategies to reduce this ‘cognitive dissonance’. One could be to neglect or reduce the importance of the conflicting information. Another strategy could be described as circumspect exposure to new information. This is achieved by, for example, avoiding newspaper articles with headlines that conflict with personal values or beliefs [16]. We did not include the theory of cognitive dissonance in the preparation of the interview guide, but we added it to the analysis after meaning condensation to contribute to the analysis of an emerging theme.

## Methods

We chose a qualitative study design because it enabled us to use and evaluate the relevance of the theoretical explanations in a continuous process. We selected screening mammography because of the extensive studies available on its benefits and harmful effects.

### Sampling and recruitment

We asked general practitioners (GPs) connected to the Research Unit for General Practice, University of Copenhagen, to briefly inform women aged 45–49 years about the study and to hand them a letter from the researchers describing the study. The selected women would be invited to participate in the breast cancer screening programme for the first time within a couple of years. The letter contained a short description of the study followed by a request for the women to contact MH if they agreed to participate. We sought to exclude women with a history of breast cancer and to include women who hold opinions on health behaviours. We favoured recruiting women through GPs who are connected to our research unit and who are familiar with their patients’ individual situations, rather than community-based recruitment.

Of the 12 women approached by the GPs, 6 contacted MH and were subsequently interviewed [18]. See Table 1 for socio-demographic details on the women who participated. No information is available for the women who failed to respond. All women were informed by phone and mail about the purpose of the study and that their participation was voluntary. Based on the provided written and verbally information, informed consent was obtained verbally by phone and preceding the interview. One week before the interview the women received postal information identical to the official invitation to attend the national breast cancer screening programme in Denmark, including the information leaflet [19].

**Table 1:** Socio-economic details for selected women

	Education	Settlement
Woman 1	Lawyer	Larger provincial town
Woman 2	Nurse aide	Smaller provincial town
Woman 3	Accountant	Capital suburb
Woman 4	Sales assistant	Smaller provincial town
Woman 5	Technical engineer	Larger provincial town
Woman 6	Accountant, early retirement	Capital

All women were aged 46–49 years

### Interviews

**Fig. 2:**
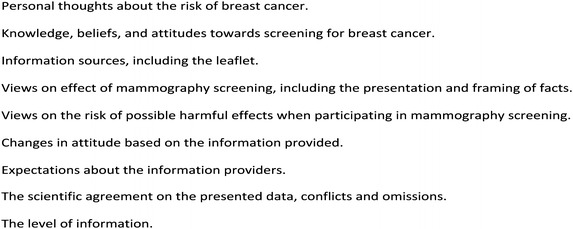
Topics covered in the interview guide. *Legend* the topics covered during the semi-structured interviews with the informants

**Fig. 3:**
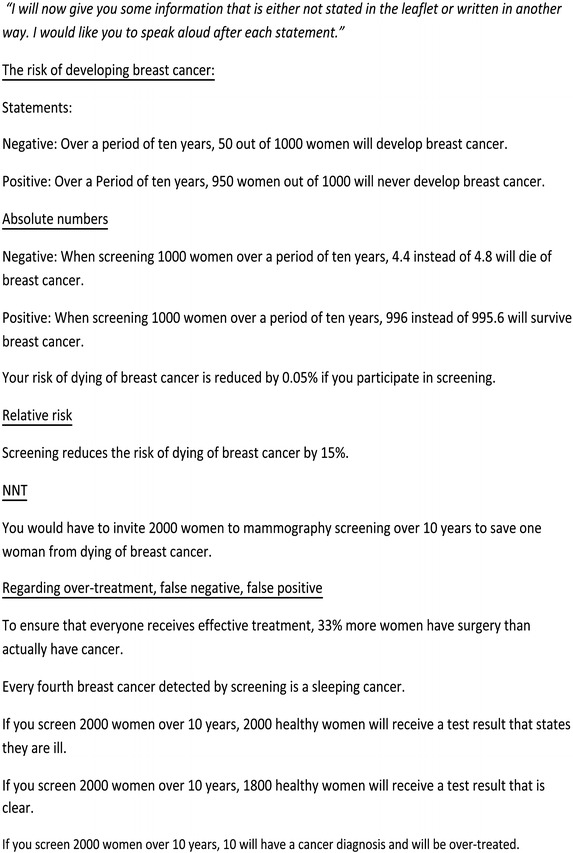
Presenting facts on Breast Cancer Screening [7]. *Legend* these statements where provided to all informants during the interview. Each statements where followed by questions to explore the informants understanding of the information as questions on changes on attitudes to the screening programme

The interviews were conducted at either the women’s homes or at their workplaces and each interview lasted approximately 90 min. The interview guide included questions on attitudes towards screening after having read the information leaflet, other sources of information, and people in the family or circle of friends who may have had an influence on the decision-making process. Inspired by Tversky, the interview guide provided the women with information about the risk of getting breast cancer and the effects of screening in both a survival frame and a mortality frame. We also presented the numbers in different formats (Fig. 2). Other questions covered the women’s knowledge of the effects of mammography screening and phenomena such as risk of side-effects, including overdiagnosis, by presenting statements about these issues framed in different ways. To optimize the effect of how changes in attitude are sensitive to framings and facts, we chose evidence based information that conflicted somewhat with the information in the official leaflet (Fig. 3). These facts were based on a Cochrane Systematic Review [7], as this was the highest level of evidence available at the time of our study design, and it pre-dates the results of The UK Independent Panel on Breast Cancer Screening.

MH conducted and audio recorded all the interviews and transcribed the material the day following the interview. All speech was transcribed but intonations were noted only if they had specific interest.

### Analysis

MH and ADG reviewed all interviews. A systematic condensation of meaning was conducted as described by Kvale [18]. Each interview was read repeatedly to get a sense of the whole and to identify themes. The researchers discussed the themes until agreement was reached and relevance according to the research questions was clarified. Meaning units were identified and condensed. These steps were followed by a cross-case analysis to summarise recurrent themes. The inductive analysis was followed by an analysis guided by TPB and the theory of cognitive dissonance.

### Role of researchers

Our research team is made up of a GP and researcher with a longstanding research interest in the psycho-social consequences of screening for breast cancer (JB) and a young doctor (MH) who was new to the field of screening and brought a particular interest in the area of doctor-patient communication and health behaviour. ADG’s main interest is doctor-patient communication in preventive care. All three are doctors and none of the women interviewed were being treated by the researchers.

### The Danish screening programme

In Denmark, women aged 50–69 are offered biannual mammography screening and they are invited by their regional health service by letter. The invitation includes a pre-booked time and date for the screening visit and an information leaflet. Therefore, women do not need referral from their GP to enter the screening programme. In cases where further examinations are needed, the regional health service contacts the women directly, bypassing the GP.

The Danish information leaflet contains information on the purpose of screening, a short description of possible benefits and unintended harmful effects, practical issues about the procedure, a description of breast cancer disease, and selected numbers about screening for breast cancer.

## Ethics

According to Danish legislation, qualitative studies are not required to seek approval from the ethics committee or the Danish Data Protection Agency. Informed consent was obtained from all participating women.

## Results

In our analysis, meaning condensation identified three themes:

- The decision-making process was dominated by the attitudes of the circle of acquaintances and to a lesser extent by the information accompanying the screening invitation.
- Information conflicting with the women’s established attitude was actively disregarded.
- The risk of overdiagnosis as a potentially harmful effect of participation in mammography screening was unknown to the women.

### The decision-making process was dominated by the attitudes of the circle of acquaintances

As part of the discussion about reasons for participating, or not, in mammography screening, we asked the women for their sources of information and their reflections on other peoples’ attitudes to screening for breast cancer. The answers revealed that the decision-making process was dominated by the attitudes prevalent in the circle of acquaintances. The information in the invitation leaflet had little influence on this process. All women except one said that they had already made their decision, despite not yet having been invited to attend the screening programme.

The women indicated different sources that influenced their decision to participate. Some valued positive experiences with mammography screening and the attitudes of close relatives.

*“My Mum has also gone to it [mammography screening], so I’ve been talking to her about it and she is comfortable with it too and thinks it’s worthwhile*”. Woman 2.

Others valued the attitudes of their friends and of peers and colleagues. All women had been made aware, through various sources, that breast cancer was a disease that was generally threatening.

### Information conflicting with the women’s established attitude was actively disregarded

Across all interviews, the interviewer explored the interaction between attitudes and the facts provided in the information leaflet. When asked to explain the advantages and disadvantages of breast cancer screening, most of the women were only able to recall one potential type of harm: the risk of “*false alarm*”. Conversely, the women were able to refer to data from the leaflet on the benefits of participating, which gave the impression of a circumspect exposure to the information in the leaflet. None of the women expressed a wish to seek out more facts, and after being provided with more information, only one woman reconsidered her decision. The other women devalued the importance of information that conflicted with their initial attitude.

This was one of the key findings in our study and can be considered as an example of cognitive dissonance. The conflict arose as the interviewer presented information such as:

*If we compare two groups: one with, the other without, screening for breast cancer, there will be no difference in the time they live.*

This contradicted the women’s expectations about screening and therefore led to contradictory conclusions.

*“It doesn´t matter if I don´t live longer, as long as I get saved from dying [from breast cancer].”*

Woman 4.

Speaking about the risk of having a false alarm, one woman stated:

*“I would be scared to death if I received a letter describing an unspecific finding on my mammogram.”* Woman 6.

Later in the interview the same woman concluded that the risk of a false alarm was fully acceptable.

The women described a possible risk that the information leaflet could interfere with the decision they had already made. They emphasised that since breast cancer is a potentially deadly disease, the information should not compromise a woman’s participation.

A few women stated that the information leaflet should be viewed as instructions on what to do, rather than information for informed decision-making.

*“It depends on what it is [which kind of health intervention]. This [the information leaflet] is just something you read before you go to that screening”.* Woman 1.

Mammography screening was considered expedient by the women in our study, and therefore not worth special consideration. Therefore demands on the accompanying information were minimal.

On the other hand one woman expressed a concern that many women were not aware of the facts surrounding the benefits and harmful effects when participating.

*“But that’s a little provoking because they do not really know what it is they are getting into”*

Woman 2.

We did not find any indication of an isolated framing effect or framing manipulation effect, whereby the decision-making process was substantially altered by how the information on breast cancer screening was framed. Instead the women seemed to seek a framing that complied with their pre-existing perceptions.

### The risk of overdiagnosis in mammography screening was unknown

One of the issues discussed during the interviews was the screening programme’s efficacy and its possible side-effects. Many of the women referred to the phrase: “*early detection leads to better treatment and saves lives*.” When speaking about the risk of side-effects, they could all imagine the risk of being anxious while waiting for the test result. This fear could be exacerbated if the woman received a letter about an unspecified finding on the mammogram. The women agreed that this risk was acceptable relative to the aim of participation. The interviewer asked how the women felt about the fact that some participants would be identified as cancer patients and offered surgery despite their cancer being non-progressive. This information clearly challenged the women, and they often asked for the question to be repeated, or they articulated counter-questions or counter-statements. The women expected a biopsy to be a way of discriminating between progressive and non-progressive cancers. Rather than using medical terminology, e.g. carcinoma in situ, the interviewer referred to sleeping cancers that might or might not wake. The women agreed that the risk of “losing a breast” due to non-progressive cancer was high, and they were unaware of this potential harm.

*“The newspapers don’t put anything about many of them not evolving.”* Woman 2.

*“… it says [the information leaflet], but not directly, that you can risk actually becoming ill.”*

Woman 3.

One woman, who had questioned her participation in the screening programme from the beginning of the interview, clearly stated her unwillingness to participate based on the information about overdiagnosis.

## Discussion

The main finding of this study is the identification of an interaction between knowledge that comes from a woman’s circle of acquaintances and the information leaflets presented by regional health services about breast cancer screening. Our findings suggest that the decision-making process relies largely on subjective norms and that information that is not compatible with women’s established attitudes is actively disregarded.

Another key finding is the lack of awareness regarding overdiagnosis of breast cancer as a result of mammography screening. This finding underlines the recommendations of the UK Independent Panel [8], and is crucial when speaking of informed consent for breast cancer screening.

Using TPB as a framework for understanding the women’s intention to participate or not in breast cancer screening, our findings suggest that the intention is strongly tied to pre-existing knowledge and attitudes adopted from family and friends. Our study reveals the importance of a woman’s circle of aquaintances in shaping attitudes and gaining knowledge. Randomised controlled trials that provided women with decision aids that helped visualise the balance between benefits vs. harmful effects found no significant difference in actual participation rates compared to women who received only basic information leaflets [20, 21]. Our study adds a further dimension to this finding, in that presenting evidence based information appears to conflict with a woman’s pre-existing perception of the balance between benefits and risks, and it kindles cognitive dissonance. This might explain the findings of the randomized trials. In this study we observed the women reducing cognitive dissonance by minimizing and devaluing the information that conflicted with their pre-existing understanding of breast cancer and mammography. Further studies should address the impact of cognitive dissonance and the intention to participate based on TPB, and not only assess the understanding of material in information leaflets.

The existence of the phenomenon of overdiagnosis and the fact that a cancer diagnosis is not definitive was new knowledge to the women who participated in our study. Further, we unexpectedly identified an unspecific resistance from the women to accept or understand the facts about overdiagnosis, in particular the lack of difference in life expectancy when comparing screened and non-screened groups. The low number of women in this study provides no indication of the prevalence of the phenomenon, but nevertheless it is noteworthy.

An Australian study focusing on the perception and view of overdiagnosis among women invited to participate in breast cancer screening also concluded that the phenomenon of overdiagnosis is hard for women to understand [10]. The Australian study and the present study hypothesise that the values regarding screening are already formed. Our study adds a further detail, in that information that conflicts with data we provided to women on subjects such as overdiagnosis comes from the circle of acquaintances, and the interaction between the two sources can be explained by TPB.

The lack of awareness among women about the harmful effects of breast cancer screening may derive from the sparse coverage in health service information leaflets [11, 12] and on the internet [22]. However, in our study we observed that women seem to strike towards an internal consistency by devaluing and not taking full note of the information leaflets. Therefore, we might question whether adding more information to the leaflets is the solution. Indications of this phenomenon of selective information seeking have been shown in an experimental design regarding colorectal screening [23]. The observation of cognitive dissonance in relation to evidence based information about screening has been described earlier by Steckelberg [24], without attracting great attention. However cognitive dissonance could explain why studies about decision aids describe change in knowledge, but no change in actual behaviour [20, 21], and should be the subject for further studies.

## Implications

Our results indicate that there are several underexplored aspects regarding the aim of achieving informed consent.

Two independent reviews on breast cancer screening have recommended improving the information material preceding participation [8, 25]. The present study also suggests that this task might be more complex than simply increasing the content of information leaflets. Even the existing information appears not to be fully understood, or even actively disregarded by women. The implications of this study are therefore twofold. The results could be used to enhance the sensitivity and accuracy of further studies, based on more quantitative strategies, such as surveys, which should include assessment of women’s perception of breast cancer screening. Further, developers of breast cancer screening information should consider more interactive information strategies addressing the problems of cognitive dissonance described in this study. In particular, overdiagnosis, which is the most harmful potential risk of participating in screening, should be addressed. This study has identified that women seem to have serious difficulty understanding the phenomenon and applying it to their own health situation. Relying solely on written information carries the risk of incomplete or biased understanding.

## Strengths and limitations

The fact that the information on breast cancer screening emanated from the University could have given the women the perception that the study’s aim was to increase participation in mammography screening. The interviewer handled this possible position by emphasising that the purpose was to focus on the women’s view and understanding of the information, and not the intervention.

The study has strength in the application of robust theory that is relevant to how information is understood and how informed consent is developed. As our study aimed at investigating the sources of information women used to shape their attitudes towards a health intervention, we found that TPB was the most relevant, since it addresses those aspects that are related to forming intentions for specific health behaviour. We included the theory of cognitive dissonance during the analytic process as the inductive analysis suggested that the women’s approach to information could be described as circumspect exposure. The theory of cognitive dissonance was integrated to optimize the understanding of our data. The grounding of our data in theoretical perspectives adds to the transferability of our findings.

The findings from a qualitative study are not thought of as facts that are applicable to the population at large, but rather as descriptions, notions, or theories applicable within a specified setting [26]. Our findings provide insight into the considerations women may face when they receive their first invitation to attend breast cancer screening. Further qualitative studies might reveal more aspects but would probably not dismiss the ones described by us. The findings of this study were represented through all informants.

The conclusions of our study are not applicable to the general population but the descriptions of influences and actual lay practices will be useful in the preparation for larger and more representative studies quantifying such aspects. Therefore we recommend our findings to future studies on the impact of women’s decision-making processes on health interventions, such as breast cancer screening.

## Conclusion

Women invited for breast cancer screening already have an expectation regarding its effect, and this influences their perception of the information provided in leaflets. Evidence based information can be actively disregarded, and if it is presented in different ways, the women will gravitate towards a presentation of the facts that does not conflict with, or that even confirms, their expectations. Women’s knowledge and attitudes come mainly from their circle of acquaintances. There is limited knowledge about overdiagnosis, and this is incompatible with the intentions of informed consent.

## References

[1] O’Neill O (2003). Some limits of informed consent. J Med Ethics.

[2] Thornton H, Edwards A, Baum M (2003). Women need better information about routine mammography. BMJ.

[3] Jørgensen KJ, Brodersen J, Hartling OJ, Nielsen M, Gøtzsche PC (2009). Informed choice requires information about both benefits and harms. J Med Ethics.

[4] Kalager M, Zelen M, Langmark F, Adami H-O (2010). Effect of screening mammography on breast-cancer mortality in Norway. N Engl J Med.

[5] Brodersen J, Jørgensen KJ, Gøtzsche PC (2010). The benefits and harms of screening for cancer with a focus on breast screening. Pol Arch Med Wewn.

[6] Brodersen J, Thorsen H (2008). Consequences of Screening in Breast Cancer (COS-BC): development of a questionnaire. Scand J Prim Health Care.

[7] Gøtzsche PC, Nielsen M. Screening for breast cancer with mammography. Cochrane Database Syst Rev. 2009;(4):CD001877. 10.1002/14651858.CD001877.pub3 19821284

[8] Independent UK Panel on Breast Cancer Screening (2012). The benefits and harms of breast cancer screening: an independent review. Lancet.

[9] Gigerenzer G, Mata J, Frank R (2009). Public knowledge of benefits of breast and prostate cancer screening in Europe. J Natl Cancer Inst.

[10] Hersch J, Jansen J, Barratt A, Irwig L, Houssami N, Howard K, Dhillon H, McCaffery K (2013). Women’s views on overdiagnosis in breast cancer screening: a qualitative study. BMJ.

[11] Slaytor EK, Ward JE (1998). How risks of breast cancer and benefits of screening are communicated to women: analysis of 58 pamphlets. BMJ.

[12] Jørgensen KJ, Gøtzsche PC (2006). Content of invitations for publicly funded screening mammography. BMJ.

[13] Ploug T, Holm S, Brodersen J (2012). To nudge or not to nudge: cancer screening programmes and the limits of libertarian paternalism. J Epidemiol Community Health.

[14] Tversky A, Kahneman D (1981). The framing of decisions and the psychology of choice. Science.

[15] Edwards AG, Naik G, Ahmed H, Elwyn GJ, Pickles T, Hood K, Playle R (2013). Personalised risk communication for informed decision making about taking screening tests. Cochrane Database Syst Rev.

[16] Ajzen I (1991). The theory of planned behavior. Organ Behav Hum Decis.

[17] Festinger L (1951). A theory of cognitive dissonance.

[18] Kvale S, Brinkmann S (2009). InterView: Introduktion Til et Håndværk.

[19] Mammografi—screening for brystkræft Invitation til mammografi. [http://www.sst.dk/publ/publ2008/plan/screening/mammografi_pjece.pdf].

[20] Mathieu E, Barratt A, Davey HM, McGeechan K, Howard K, Houssami N (2007). Informed choice in mammography screening: a randomized trial of a decision aid for 70-year-old women. Arch Intern Med.

[21] Mathieu E, Barratt AL, McGeechan K, Davey HM, Howard K, Houssami N (2010). Helping women make choices about mammography screening: an online randomized trial of a decision aid for 40-year-old women. Patient Educ Couns.

[22] Jørgensen KJ, Gøtzsche PC (2004). Presentation on websites of possible benefits and harms from screening for breast cancer: cross sectional study. BMJ.

[23] Steckelberg A, Kasper J, Mühlhauser I (2007). Selective information seeking: can consumers’ avoidance of evidence-based information on colorectal cancer screening be explained by the theory of cognitive dissonance?. Ger Med Sci.

[24] Steckelberg A, Kasper J, Redegeld M, Mühlhauser I (2004). Risk information–barrier to informed choice? A focus group study. Soz Praventivmed.

[25] Biller-Andorno N, Jüni P (2014). Abolishing mammography screening programs? A view from the Swiss Medical Board. N Engl J Med.

[26] Malterud K (2001). Qualitative research: standards, challenges, and guidelines. Lancet.

